# Supplementary material for: Silencing cryptic specialized metabolism in Streptomyces by the nucleoid-associated protein Lsr2
Source: eLife. 2019 Jun 19;8:e47691. doi: 10.7554/eLife.47691 (PMC6584129; doi:10.7554/eLife.47691)
Supplement: Supplementary file 5. [file elife-47691-supp5.docx]

| **Supplementary File 5. Highly abundant (intensities >10^5^) unique or significantly upregulated (>5 fold) compounds**  *Red: new compounds relative to previous days* | | |
| --- | --- | --- |
|  |  |  |
| **3 Day Compound List** |  |  |
| ***Unique Compounds*** |  |  |
| ***m/z*** | **Rt (min)** |  |
| 293.08 | 3.28 |  |
| 721.31 | 8.6 |  |
| 606.28 | 8.7 |  |
| 281.07 | 9.14 |  |
| 172.02 | 9.22 |  |
|  |  |  |
| ***Significantly Upregulated Compounds*** | |  |
| ***m/z*** | **Rt (min)** |  |
| 172.09 | 3.81 |  |
| 186.11 | 5.03 |  |
| 546.49 | 16.23 |  |
|  |  |  |
| **4 Day Compound List** |  |  |
| ***Unique Compounds*** |  |  |
| ***m/z*** | **Rt (min)** |  |
| 293.08 | 3.26 |  |
| 606.28 | 8.76 |  |
| 281.07 | 9.12 |  |
| 668.22 | 11.3 |  |
|  |  |  |
| ***Significantly Upregulated Compounds*** | |  |
| ***m/z*** | **Rt (min)** |  |
| 247.12 | 3.79 |  |
| 172.09 | 3.79 |  |
| 186.1 | 5 |  |
|  |  |  |
| **5 Day Compound List** |  |  |
| ***Unique Compounds*** |  |  |
| ***m/z*** | **Rt (min)** |  |
| 293.08 | 3.25 |  |
| 606.2 | 8.7 |  |
| 281.07 | 9.11 |  |
| 172.01 | 9.17 |  |
|  |  |  |
| ***Significantly Upregulated Compounds*** | |  |
| ***m/z*** | **Rt (min)** |  |
| 247.12 | 2.12 |  |
| 172.09 | 3.8 |  |
| 614.27 | 4.8 | Ferrioxamine B |
| 186.11 | 5.02 |  |
|  |  |  |
