# Supplementary material for: Silencing cryptic specialized metabolism in Streptomyces by the nucleoid-associated protein Lsr2
Source: eLife. 2019 Jun 19;8:e47691. doi: 10.7554/eLife.47691 (PMC6584129; doi:10.7554/eLife.47691)
Supplement: Supplementary file 6. [file elife-47691-supp6.docx]

**Supplementary File 6. Putative identification of the most abundant, differentially expressed volatile compounds by wild type and Δ*lsr2* strains of *S. venezuelae.***

| **Compound # - WT** | **Putative ID #1 (Match Score)** | **Putative ID #2 (Match Score)** | **Putative ID #3 (Match Score)** |
| --- | --- | --- | --- |
| 1 | Terpinen-4-ol **(823)** | Terpinen-4-ol **(810)** | Terpinen-4-ol **(800)** |
| 2 | Germacrene D **(845)** | Copaene **(810)** | α-amorphene **(810)** |
| 3 | β-elemene **(837)** | γ-elemene **(818)** | β-elemene **(813)** |
| 4 | δ-elemene **(755)** | Bicyclo[3.1.0]hexane, 6-isopropylidene-1-methyl- **(753)** | δ-elemene **(737)** |
| 5 | 4-methylene-2,8,8-trimethyl-2-vinylbicylo[5.2.0]nonane **(833)** | α-bulnesene **(832)** | α-selinene **(822)** |
| 6 | α-campholenal **(758)** | 6-camphenol **(749)** | α-campholenal **(744)** |
| 7 | α-amorphene **(785)** | γ-muurolene **(780)** | γ-muurolene **(779)** |
| 8 | β-elemene **(824)** | β-elemene **(821)** | γ-elemene **(813)** |
| 9 | 6-epi-shyobunol **(751)** | Viridiflorol **(734)** | Epiglobulol **(734)** |
| 10 | 4,4-dimethyl-2-cyclohexene-1-ol **(716)** | Geosmin **(713)** | trans-1,10-Dimethyl-trans-9-decalinol **(712)** |
| 11 | 6-camphenol **(781)** | Bicyclo[2.2.1]heptane-3-methylene-2,2-dimethyl-5-ol acetate **(777)** | 1,7,7-Trimethylbicyclo[2.2.1]hept-  5-en-2-ol **(766)** |
| 12 | *o*-cymene **(888)** | *p-*cymene **(887)** | *o*-cymene **(885)** |
| 13 | β-thujene **(759)** | α-thujene **(748)** | α-thujene **(746)** |
| 14 | α-phellandrene **(782)** | α-thujene **(772)** | 4-carene **(767)** |
| 15 | 5-isopropyl-6-methyl-hepta-3,5-dien-2-ol **(720)** | Cyclohexene, 1-acetyl-2-(1-hydroxyethyl)- **(711)** | - |

| **Compound # - Δ*lsr2*** | **Putative ID #1**  **(Match Score)** | **Putative ID #2**  **(Match Score)** | **Putative ID #3**  **(Match Score)** |
| --- | --- | --- | --- |
| 1 | Isopropanol **(922)** | Isopropanol **(878)** | Isopropanol **(878)** |
| 2 | Acetone **(978)** | Acetone **(969)** | Acetone **(964)** |
| 3 | Benzaldehyde **(951)** | Benzaldehyde **(932)** | Benzaldehyde **(926)** |
| 4 | 2-butanone **(918)** | 2-butanone **(917)** | 2-butanone **(913)** |
| 5 | Butyl acetate **(885)** | Butyl acetate **(882)** | Butyl acetate **(857)** |
| 6 | Acetoin **(877)** | Acetoin **(869)** | L-lactic acid **(776)** |
| 7 | 3-methyl-2-butanone **(905)** | 3-methyl-2-butanone **(883)** | 3-methyl-2-butanone **(865)** |
| 8 | Butanethioic acid **(788)** | Methyl isobutanethioate **(764)** | Butanethioic acid **(733)** |
| 9 | Benzyl alcohol **(917)** | Benzyl alcohol **(900)** | Benzyl alcohol **(895)** |
| 10 | 3-methyl-3-buten-2-one **(934)** | 1-cyclopropylethanone **(912)** | 3-methyl-3-buten-2-one **(896)** |
| 11 | sec-Amyl acetate **(908)** | sec-Amyl acetate **(900)** | sec-Amyl acetate **(867)** |
| 12 | 3-methyl-2-butanol **(795)** | 3-methyl-2-butanol **(786)** | 3-methyl-2-butanol **(779)** |
