# Supplementary material for: Silencing cryptic specialized metabolism in Streptomyces by the nucleoid-associated protein Lsr2
Source: eLife. 2019 Jun 19;8:e47691. doi: 10.7554/eLife.47691 (PMC6584129; doi:10.7554/eLife.47691)
Supplement: Supplementary file 7. [file elife-47691-supp7.docx]

**Supplementary File 7. Bacterial strains, plasmid and cosmids used in this work**

| **Strain** | | **Genotype/characteristics/use** | | **References** | |
| --- | --- | --- | --- | --- | --- |
| ***Streptomyces*** | |  | |  | |
| *S. venezuelae* ATCC 10712 | Wild type | |  | |  |
| E327 | *S. venezuelae* *lsr2::aac(3)IV* | | This study | |  |
| E327A | *S. venezuelae* Δ*lsr2* | | This study | |  |
| E328 | *S. venezuelae* Δ*lsrL::aac(3)IV* | | This study | |  |
| *S. coelicolor* M145 | Wild type | | (1) | |  |
| Wild isolate WAC4718 |  | | Gift from G. Wright | |  |
| Wild isolate WAC5514 |  | | Gift from G. Wright | |  |
| Wild isolate WAC6377 |  | | Gift from G. Wright | |  |
| Wild isolate WAC7072 |  | | Gift from G. Wright | |  |
| Wild isolate WAC7520 |  | | Gift from G. Wright | |  |
| ***Escherichia coli*** |  | |  | |  |
| DH5α | Routine cloning | | (2) | |  |
| SE DH5α | Highly-competent (Subcloning | | Invitrogen | |  |
|  | Efficiency™) DH5α cells | |  |  |  |
| ET12567 | *dam*, *dcm*, *hsdS*, *cat*, *tet*; carries *trans-*mobilizing plasmid pUZ8002 | | (3, 4) | |  |
| Rosetta 2 | Protein overexpression host with pRARE2 which supplies ‘rare’ tRNAs | | Novagen | |  |
| **Plasmids and Cosmids** |  | |  | |  |
| Cosmid 1-C1 | *S. venezuelae* cosmid carrying *lsr2* | | Gift from M. Buttner | |  |
| Cosmid 4E19 | *S. venezuelae* cosmid carrying *lsrL* | | Gift from M. Buttner | |  |
| Cosmid Sv-3-D04 | *S. venezuelae* cosmid carrying *sven_5106-5107* | | Gift from M. Buttner | |  |
| pIJ82 | Integrative cloning vector; *ori* pUC18, *hyg*, *oriT*, RK2, *int* ФC31, *attP* ФC31 | | Gift from H. Kieser | |  |
| pMC112 | pIJ82 carrying *lsr2* | | This study | |  |
| pCR-2.1 TOPO | *E. coli* cloning vector | | ThermoFisher Scientific | |  |
| pMC116 | TOPO carrying wild type *sven_5105-5107* (58% GC in intergenic region between *sven_5106* and *5107*) | | This study | |  |
| pMC117 | TOPO carrying *sven_5105-5107* with 63% GC region between *sven_5106* and *5107* | | This study | |  |
| pMC118 | TOPO carrying *sven_5105-5107* with 70% GC region between *sven_5106* and *5107* | | This study | |  |
| pRT801 | Integrative cloning vector: *aac(3)IV, oriT, int* ФBT1, *attP* ФBT1 | | (5) | |  |
| pMC119 | pRT801 carrying wild type *sven_5105-5107* | | This study | |  |
| pMC120 | pRT801 carrying *sven_5105-5107* with 63% GC | | This study | |  |
| pMC121 | pRT801 carrying *sven_5105-5107* with 70% GC | | This study | |  |
| pIJ10706 | Integrative cloning vector; identical to pIJ82, apart from the promoter driving the expression of *hyg* | | (6) | |  |
| pMC113 | pIJ10706 carrying *lsr2-3×Gly-3×FLAG* | | This study | |  |
| pET15b | Overexpression of N-terminally His_6_-tagged proteins | | Novagen | |  |
| pMC114 | pET15b carrying *lsr2*for overexpression with an N-terminal His6-tag | | This study | |  |
| pCRISPomyces-2 | Cas9-carrying plasmid for CRISPR-mediated sequence deletion | | (7) | |  |
| pMC115 | pCRISPomyces-2 carrying the guide RNA and editing template sequence for deleting *SVEN_6229* | | This study | |  |
| pSET152 | Integrating cloning vector, identical to pIJ82, only carrying the *aac(IV)3* resistance gene (apramycin) instead of *hyg* | | (8) | |  |
| pIJ12551 | pSET152 with *ermE** promoter in multiple cloning site | | (9) | |  |
| pMC109 | *lsr2-*R82A mutant variant cloned downstream of *ermE** promoter in pIJ12551 | | This study | |  |

1. Kieser T, Bibb MJ, Buttner MJ, Chater KF, Hopwood DA (2000) *Practical* Streptomyces *Genetics* (The John Innes Foundation, Norwich, U.K.).

2. Hanahan D (1985) Techniques for transformation of *E. coli*. *DNA Cloning - A Practical Approach Vol 1*:109–135.

3. MacNeil DJ, et al. (1992) Analysis of *Streptomyces avermitilis* genes required for avermectin biosynthesis utilizing a novel integration vector. *Gene* 111(1):61–68.

4. Paget MSB, Chamberlin L, Atrih A, Foster SJ, Buttner MJ (1999) Evidence that the extracytoplasmic function sigma factor σ^E^ is required for normal cell wall structure in *Streptomyces coelicolor* A3(2). *J Bacteriol* 181(1):204–211.

5. Gregory MA, Till R, Smith MCM (2003) Integration site for *Streptomyces* phage φBT1 and development of site-specific integrating vectors. *J Bacteriol* 185:5320–5323.

6. Foulston LC, Bibb MJ (2010) Microbisporicin gene cluster reveals unusual features of lantibiotic biosynthesis in actinomycetes. *Proc Natl Acad Sci U S A* 107(30):13461–13466.

7. Cobb RE, Wang Y, Zhao H (2015) High-efficiency multiplex genome editing of *Streptomyces* species using an engineered CRISPR-Cas system. *ACS Synth Biol* 4:723–728.

8. Bierman M, et al. (1992) Plasmid cloning vectors for the conjugal transfer of DNA from *Escherichia coli* to *Streptomyces* spp. *Gene* 116(1):43–49.

9. Sherwood EJ, Hesketh AR, Bibb MJ (2013) Cloning and analysis of the planosporicin lantibiotic biosynthetic gene cluster of *Planomonospora alba*. *J Bacteriol* 195(10):2309–2321.
