# Supplementary material for: Silencing cryptic specialized metabolism in Streptomyces by the nucleoid-associated protein Lsr2
Source: eLife. 2019 Jun 19;8:e47691. doi: 10.7554/eLife.47691 (PMC6584129; doi:10.7554/eLife.47691)
Supplement: Supplementary file 8. [file elife-47691-supp8.docx]

| **Supplementary File 8. Oligonucleotides and synthetic DNA used in this study** | |  |
| --- | --- | --- |
| **Gene knockout** | **Sequence (5'-3)*** | **Use** |
| Sven3225disruptF | TTTCCGCGTACTGCATTTTCGATGAAAGGAAATCCGGTGATTCCGGGGATCCGTCGACC | Replace *lsr2* with an apramycin resistance cassette |
| Sven3225disruptR2 | CCAGCAGCTGTCGCCTCAGCGGTTCGCGTCCTCGTAGGCTGTAGGCTGGAGCTGCTTC | Replace *lsr2* with an apramycin resistance cassette |
| Sven3225F2 | GGTATTCTGCCTGACCTTCG | Confirm deletion of *lsr2* |
| sven3225R2 | GTGGCGCCGAGGAACTACC | Confirm deletion of *lsr2* |
| Sven3832disruptF | TGCACCGGGACACGCTGAAACAATTGCCAGGGCTTCACAATTCCGGGGATCCGTCGACC | Replace *lsrL* with an apramycin resistance cassette |
| Sven3832disruptR | AAGCGGGTCTACGAGGCCTTCCGCGCCGCGAGTTGAGCGTGTAGGCTGGAGCTGCTTC | Replace *lsrL* with an apramycin resistance cassette |
| sven3832F3 | CCCTCAGCGTACCTGTCTCC | Confirm deletion of *lsrL* |
| sven3832R3 | GAGCACCACTCTACCTGATGC | Confirm deletion of *lsrL* |
| Sven6229 GuideF | AAACGAAGACCGAGCGTCTGGTCA | Guide RNA for CRISPR deletion within *6229* |
| Sven6229 GuideR | ACGCTGACCAGACGCTCGGTCTTC | Guide RNA for CRISPR deletion within *6229* |
| Sven6229 UpF | CATAG**TCTAGA**GGGAAGACCCAGTTCCCCTA | Generate upstream editing template for CRISPR deletion in *6229* |
| Sven6229 UpR | GATGAGCTCGAACTCCTGGA | Generate upstream editing template for CRISPR deletion in *6229* |
| Sven6299 DownF | TCCAGGAGTTCGAGCTCATCATGCCGAAGGCCTACGACTAC | Generate downstream editing template for CRISPR deletion in *6229* |
| Sven_6229 DownR | GCACT**TCTAGA**GAAGAACGTGGGGACCAGTG | Generate downstream editing template for CRISPR deletion in *6229* |
| 6229 Con Rev | CGACCAGGTAGAAGACCAGAC | Confirmation of CRISPR deletion |
|  |  |  |
| **Complementation** | **Sequence (5'-3)** |  |
| Sven3225F | TTCCTATGACGAGGGAGTCG | Complementation of *lsr2*deletion |
| Sven3225R | GAATGGGGCGGTATCTCG | Complementation of *lsr2*deletion |
|  |  |  |
| ***5105-07* cloning and mutagenesis** | **Sequence (5'-3)**** |  |
| Sven5105_5107F | ATAT**GGATCC**CTTCGAACTCGACCGGACCGG | Cloning *sven_5105-5107* |
| Sven5105-5107R | ATAT**GGATCC**CCGGTGATCCGGATCCCCAG | Cloning *sven_5105-5107* |
| Sven5106-5107F | GGCTAGCAGCACGAAAAACC | Cloning mutant sequence into *sven_5106-7* |
| Sven5106-5107R | AGCCTAGGCGCAGGCTCCGAA | Cloning mutant sequence into *sven_5106-7* |
| 63% GC content | CGACCAGCCTAGGCGCAGGCTCCGAAGCGTGGGTGAAATCCCTTTTCCCGGCTCGGTCGAGTTCTCGTGCACGGGGACTGCAGTTGCTGTTCCACGTTGCTCTCGGCAGCGTTGTCCAGGGCCGCGAAGTGCCCGCTGACCTGGGGGTGTTGTGCCCGCTGGGCGAGTCCTCCTGGGCTGTAGGCAAAGATCAACTCGGTTTTTCGTGCTGCTAGCCTGACCCGGATTTTGCACAGCCA | Replacing wild type 58% GC sequence with 63% GC sequence between *sven_5106* and *sven_5107* |
| 64% GC content | CGACCAGCCTAGGCGCAGGCTCCGAAGCGTGGGTGAAATCCCTTTTCCCGGCTCGGTCGATTTCTCGTGCACGGGGAATTCAGGGGCGGGGCCCCGTTTCGCGCGGCCGCGGGGGCCATGGCCGCGAAGTGCCCGCTGAACTGGGGGTTTTGTGCCAGCTGGGCGATTCATCCTGGTCTGTAGGCAAAGATCAACTCGGTTTTTCGTGCTGCTAGCCTGACCCGGATTTTGCACAGCCA | Replacing wild type 58% GC sequence with 64% GC sequence between *sven_5106* and *sven_5107* |
| 70% GC content | CGACCAGCCTAGGCGCAGGCTCCGAAGCGTGGGTGAAATCCCTTTTCCCGGCTCGGTCGAGTGCTCGTGCACGGGGACGGCAGGTGCGGGTCCACGGTGCGCTCGGCAGCGTGGGCCAGGGCCGCGCAGTGCCCGCTGACCTGGGGGGGTGGTGCCCGCTGGGCGCGGCCTCCGGGGCTGTAGGCAAAGATCAACTCGGTTTTTCGTGCTGCTAGCCTGACCCGGATTTTGCACAGCCA | Replacing wild type 58% GC sequence with 70% GC sequence between *sven_5106* and *sven_5107* |
|  |  |  |
| **EMSA** | **Sequence (5'-3)** |  |
| emsa0506F | GGAATTCATCCATGGTGTGG |  |
| emsa0506R | GCCCGAAGAATCGTTTAGG |  |
| emsa5092F | CGACGATGCTGGTGTTCC |  |
| emsa5092R | ATTCCTCGGTTGACAATTCG |  |
| emsa5130F | AACTCGGATCAGCATTATTGG |  |
| emsa5130R | AGTTTCCTTTCAGCGTTTCG |  |
| emsa5133F | GGTGGATTCCGTAGTCATGG |  |
| emsa5133R | CGAGAATTCGAGAAACAACG |  |
| emsa6217F | GCATTCCTGTCCTGTTCTCC |  |
| emsa6217R | GGCAATTCTGCATAACTCTCG |  |
| svr3556F | ATATCCTCTAGAGGAGCGACTGGATGTGGAC |  |
| svr3556R | ATATCCGGTACCCCAAGGAAGAGAACAGCTTCCC |  |
|  |  |  |
| **RT-PCR primers** | **Sequence (5'-3)** |  |
| RT_0493F2 | ACCCGGATTCCTCCTATTCC |  |
| RT_0493R2 | AGGAAACGCCGTTCTATCG |  |
| RT_0514F | GAGAACGAACCGGGAGGTC |  |
| RT_0514R | CGATCTGGAGGACGAAGACG |  |
| RT_5135F2 | GGCGGCGTCTACTTCAGC |  |
| RT_5135R2 | AGGAGCAGTCCGTTCTCG |  |
| RT_5965F | GGTGAGGGCAAGGAGATCG |  |
| RT_5965R | GTGACCGTCGACTTCACCTG |  |
| RT_6216F | ACCGAAACCCTGACGTACAC |  |
| RT_6216R | GAGCAGGGTGACGATGAGC |  |
| RT_6264F | GTGATGACATCGACTCCGGG |  |
| RT_6264R | GGTAGCCGGCCGAGTTGTA |  |
| 5106F | ACGACGAGACGATGATCAGG |  |
| 5106R | ATCCGGATGTCCAGGAGGA |  |
| rpoBF | TCGACCCTTCGGCAACCGC |  |
| rpoBF | GCGCTCCATACGGGCGAGAC |  |
| SVEN4987F | ATATGGTACCGATCTGGAACGGCATCCAGG | Confirm no contaminating DNA in RNA samples |
| SVEN4987R | TATTCTAGACGCGAGGTCCTTGTTCTTGG | Confirm no contaminating DNA in RNA samples |
| HrdbF | CCGTTTCCATCGTTCCGAGA | Confirm no contaminating DNA in RNA samples |
| HrdbR | ATCTGCCCATCAGCCTTTCC | Confirm no contaminating DNA in RNA samples |
|  |  |  |
| **Lsr2 overexpression** | **Sequence (5'-3')*** |  |
| NdeISven3225F | GGGTGC**CATATG**GCACAGAAGGTTCAGGTCCTT |  |
| BamHISven3225R | ATAT**GGATCC**CCTGTCTGGTGCGTCTCG |  |
|  |  |  |
| **ChIP-qPCR** | **Sequence (5'-3')** |  |
| 0926F | CAGGCGATATCCCGTCAGTG |  |
| 0926R | GTGCACACCCCCTAGAAAGA |  |
| 5105F | TAGTACCGGCACTCGTCCAT |  |
| 5105R | TACGCGAAGATCAGGATCGG |  |
|  |  |  |
| **R82A variant** | **Sequence (5′-3′)*** |  |
| R82ASven3225F | GACACCGCGAAGATCGCCGCGTGGGCCAAGGAC |  |
| R82ASven3225R | GTCCTTGGCCCACGCGGCGATCTTCGCGGTGTC |  |
| NdeISven3225F | GGGTGC**CATATG**GCACAGAAGGTTCAGGTCCTT |  |
| PacI3225R | GGGTGC**TTAATTAA**CCTGTCTGGTGCGTCTCG |  |

* Restriction enzyme sequences are bolded and underlined

** Mutations are indicated in red text (changing AT nucleotides to GC nucleotides)
